# ‘It depends entirely on the nature of those supports’: Community perceptions of the appropriateness of early support services for autistic children

**DOI:** 10.1177/13623613241302372

**Published:** 2024-12-04

**Authors:** Rhylee Sulek, Chris Edwards, Ruth Monk, Lee Patrick, Sarah Pillar, Andrew JO Whitehouse, Hannah Waddington

**Affiliations:** 1 Griffith University, Australia; 2 The Kids Research Institute Australia, Australia; 3 Autism Spectrum Australia, Australia; 4 Autism New Zealand, New Zealand; 5 University of Auckland, New Zealand; 6 Te Herenga Waka–Victoria University of Wellington, New Zealand

**Keywords:** autism, co-production, early support services, neurodiversity, reflexive thematic analysis

## Abstract

Autistic advocates emphasise the need for neurodiversity-affirming and strengths-based approaches to support services; however, little is known about broader community perspectives regarding the appropriateness of offering early support services to autistic children. This co-designed mixed-methods study employed surveys to gather insights from 253 participants in Australia and New Zealand, including autistic adults, parents, and professionals. Participants shared views on the appropriateness of early support services for autistic children. About half of participants indicated that it was appropriate to provide early support services, while the other half indicated that it depended on the nature of those support services. Reflexive thematic analysis resulted in three overarching themes which explain these views. ‘They are children first, after all’ emphasises the importance of preserving childhood experiences and involving children in decision-making. ‘We shouldn’t be aiming to fix the child’ underscores the need for support services to align with neurodiversity-affirming approaches. Finally, ‘Supports are beneficial’ highlights the perceived positive impact that early, individualised support services can provide for autistic children. These findings predominantly signal a shift away from medicalised models towards a neurodiversity-affirming approach across participant groups.

**Lay abstract:**

We do not know much about what support services people think are okay for young autistic children. This study was a survey of 253 people. We asked autistic adults, parents, and professionals from Australia and New Zealand whether they thought it was okay to provide support services to autistic children. About half the people who shared their thoughts said it was okay to provide support services to autistic children and the other half said it depended on what the support service was like. They had three main ideas about whether support services were okay or not. The first one is that we should remember that these autistic children are children first, so we need to keep their childhood experiences in mind and let them have a say in decisions. The second is that we should not try to ‘fix’ the child, but instead, use supports that respect and understand the unique ways the child thinks. The final idea is that early, personalised help is good for autistic children and can make a positive difference in their lives. This study suggests that we should focus on what each child needs, think about how children can join in, and provide help in ways that respect autistic children.

Autistic individuals may benefit from receiving support services throughout the lifespan that aim to enhance learning, participation, and well-being. Both the autistic community (i.e. autistic individuals themselves) and the broader autism community (i.e. family members and other individuals who support autistic individuals) generally find support services for autistic people acceptable, as long as they are reasonable, necessary, and affirming (Australian Government, 2022; UK Parliament, 2020). The Australian Government recently recognised improving access to early support services as a key priority (Australian Government, 2022). Similarly, the Aotearoa New Zealand Autism Guideline emphasises access to high-quality support as soon as a need is identified (Whaikaha – Ministry of Disabled People and Ministry of Education, 2022).

There is a wide variety of early support services (sometimes called ‘therapeutic services’ or ‘interventions’). These have been categorised into eight broad approaches: behavioural, developmental, naturalistic developmental behavioural interventions [NDBI], TEACCH, sensory-based, animal-assisted, cognitive behavioural therapy, and technology-based support services (Sandbank et al., 2023). Some support services do not fall into these categories and have been categorised as ‘other’ (Sandbank et al., 2023). These approaches differ based on many characteristics, including the theoretical premise for how they are proposed to work, and the ways in which they are delivered including the intensity of support, the person who delivers the support service (e.g. parents, therapists), and the settings in which they are delivered (Sandbank et al., 2023). Each of these approaches further vary in the extent to which they are both appropriate for, and effective in, addressing the goals and outcomes prioritised by children and their families when accessing early support services.

Many professionals, researchers, and organisations stress the importance of providing support during the child’s first years of life (e.g. 0–3 years), framing these years as a critical developmental period (Bent et al., 2023; Edwards et al., 2017). This concept is often, in turn, embraced by parents of autistic children who access such support services. However, in line with the growing neurodiversity movement, some autistic people, family members, professionals, and members the broader community have begun calling into question the appropriateness of some of these early support services (Dawson et al., 2022; Schuck et al., 2022; Waddington et al., 2024). A neurodiversity perspective understands autism as a brain-based difference, recognising an individual’s unique strengths, challenges, and support needs, rather than as a deficit or disorder to be treated (Bottema-Beutel et al., 2021; Kapp et al., 2013; Pellicano & den Houting, 2022). Within the neurodiversity movement there is an emphasis on improving quality of life for autistic children and their families consistent with broader conceptual frameworks which define what it is to have a ‘good life’ (Nussbaum, 2000; Pellicano et al., 2022; Pellicano & Heyworth, 2023). This replaces a historical focus on reducing ‘deficits’ associated with autism. As such, services should focus on supporting autistic children to ‘thrive’ or ‘flourish’ on their own terms, facilitated by the broader contexts (e.g. family, school, community) in which they are embedded (Nussbaum, 2000; Pellicano & Heyworth, 2023).

Proponents of the neurodiversity approach have criticised some early support services, for example, certain behavioural approaches and NDBIs, for their goals, procedures, and outcomes, suggesting that they do not promote autistic quality of life (Autistic Self Advocacy Network, 2021; Leaf et al., 2022; Schuck et al., 2022). Critics argue that some support services include a desire to ‘normalise’ autistic children by teaching them to hide their autistic traits and that they do not focus on teaching and promoting meaningful life skills, self-determination, and self-esteem (Autistic Self Advocacy Network, 2021). The intensive provision of many hours of support is argued to cause the autistic child to miss out on other important life activities. The most severe criticisms relate to the use of aversive punishment such as electric shocks; the use of restraint and seclusion; and forcing or coercing children to ‘comply’ regardless of their lack of assent/consent (Autistic Self Advocacy Network, 2021; Sandoval-Norton et al., 2019). In two small studies, autistic adults identified that the use of support services with some of these characteristics in their childhoods caused significant harm or trauma and that any gain in skills such as communication was outweighed by the significant negative effects on their mental health (Anderson, 2023; McGill & Robinson, 2020).

In response to the aforementioned criticisms, some advocates, researchers, and practitioners have sought to identify ways to improve support services to better address these concerns and move towards more neurodiversity-affirming approaches. These adaptations have included a greater focus on supporting the development of meaningful life skills, using preferred communication methods, and improving quality of life (Dawson et al., 2022; Pellicano & Heyworth, 2023; Schuck et al., 2022). Suggestions for positive reframing of autism have also included the use of autistic preferred terminology and a focus on the child’s strengths and inherent value (Bottema-Beutel et al., 2021; Monk et al., 2022). Finally, advocates and researchers have emphasised the importance of valuing autistic voices by including autistic people in the design of support services and allowing autistic children to decide on their own support goals, as much as possible (den Houting, 2019; Leaf et al., 2022; Schuck et al., 2022).

Despite growing criticisms of the purpose and methods of certain early support services, there have been few scientific studies in this area. The purpose of this study was, therefore, to examine the perspectives of people from the autistic and broader autism communities regarding the appropriateness of providing early support services to autistic children.

## Method

### Design

This study combines data from two projects with the primary aims of investigating participant perspectives on the goals (Waddington et al., 2024) and outcomes (Sulek et al., 2024), respectively, of early support services. Both projects were co-design by teams of autistic and non-autistic researchers. This analysis employed a concurrent mixed-methods design, weighted towards qualitative analysis (Johnson & Christensen, 2020). The data analysed for this project pertained to a single survey question regarding participants’ perspectives of the appropriateness of early support services with quantitative and qualitative response options (see Measures section for exact wording). In line with the design, qualitative responses were designed to *explain* quantitative responses regarding the perceived appropriateness of early support services (Johnson & Christensen, 2020). For detailed information on the methodology of the broader studies, see (Sulek et al., 2024; Waddington et al., 2024).

### Participants and procedure

The studies were conducted with approval from the relevant institutions, with further approval sought to combine datasets. Individuals were eligible to participate in either study if they: (1) were at least age 16 (Sulek et al., 2024) or 18 (Waddington et al., 2024), (2) lived in Australia or New Zealand, and (3) were an autistic adult, a parent/caregiver of an autistic child, and/or a practitioner providing support services to autistic children. Participants were recruited via social media and the professional networks of the study authors, who were also encouraged to share recruitment materials among their own contacts. Both surveys were accessed online, with participants first reviewing study information before consenting to participate.

In this analysis, data were only retained for those who provided a qualitative response regarding the appropriateness of providing early support services to autistic children. Of 326 participants in Waddington et al. (2024) data for 179 (54.9%) are included in this study, and of 181 participants in Sulek et al. (2024), the data or 74 (40.8%) are included in this study. Demographic information for the combined 253 participants is presented in Table 1. For the combined sample, approximately half the participants (48.2%) identified as parents, followed by autistic individuals (40.3%), and practitioners (32.4%), with 52 participants (20.5%) indicating they identified across more than one participant group. The majority of participants identified as female (84.9%) and had either European Australian (41.1%) or European New Zealand (40.3%) ethnicities. Approximately half of participants resided in New Zealand (54.2%) with the rest residing in Australia. This was an educated sample as the majority of participants held either an undergraduate (35.2%) or post-graduate degree (33.6%).

**Table 1.** Participant demographics.

	All sample (*N* = 253)	Dataset A (*n* = 74)	Dataset B (*n* = 179)
Group			
Autistic only	58 (22.9)	20 (27.0)	38 (21.2)
Autistic parent	28 (11.1)	8 (10.8)	20 (11.2)
Autistic practitioner	7 (2.8)	6 (8.1)	1 (0.6)
Autistic parent and practitioner	9 (3.6)	4 (5.4)	5 (2.8)
Parent only	85 (33.6)	16 (21.6)	69 (38.5)
Parent and practitioner	8 (3.2)	3 (4.1)	5 (2.8)
Practitioner only	58 (22.9)	17 (23.0)	41 (22.9)
Country			
Australia	116 (45.8)	65 (87.8)	51 (28.5)
New Zealand	137 (54.2)	9 (12.2)	128 (71.5)
Gender			
Female	215 (84.9)	66 (89.2)	149 (83.2)
Male	25 (9.9)	5 (6.8)	20 (11.2)
Non-binary	11 (4.3)	4 (5.4)	7 (3.9)
Other	4 (1.6)	1 (1.4)	3 (1.7)
Ethnic group			
New Zealand European	102 (40.3)	9 (12.2)	93 (51.9)
European Australian	104 (41.1)	61 (82.4)	43 (24.0)
Māori	26 (10.2)	1 (1.4)	25 (13.9)
Aboriginal	1 (0.4)	1 (1.4)	-
Other (inc. Tongan, Samoan, Vietnamese, Indian, Chinese, Japanese)	42 (16.6)	8 (10.8)	34 (19.0)
Prefer not to say	2 (0.8)	1 (1.4)	1 (0.6)
Education			
Primary/intermediate school	2 (0.8)	-	2 (1.1)
College/high school	31 (12.2)	9 (12.2)	22 (12.3)
Trade/technical/vocational training	29 (11.4)	9 (12.2)	20 (11.2)
Bachelor’s/undergraduate university degree	89 (35.2)	26 (35.1)	63 (35.2)
Postgraduate university degree	85 (33.6)	28 (37.8)	57 (31.8)
Other	16 (6.3)	2 (2.7)	14 (7.8)
Prefer not to say	1 (0.4)	-	1 (0.6)
Do you believe it’s appropriate to provide early support services?			
Yes	127 (50.2)	29 (39.2)	98 (54.7)
Only some/it depends	122 (48.2)	45 (60.8)	77 (43.0)
No	2 (0.8)	-	2 (1.1)
Don’t know	1 (0.4)		1 (0.6)
Prefer not to say	1 (0.4)		1 (0.6)

## Measures

The included data, drawn from the broader studies, relate to participant demographics and responses to the following question: *Do you personally believe that it is okay to provide early supports to* (a) *preschool aged children* (Waddington et al., 2024) OR (b) *autistic children (aged between 0 and 12* *years)* (Sulek et al., 2024)? Response options included: ‘*yes’*, ‘*no’*, ‘*it depends on the nature of supports*’, and ‘*prefer not to say*’. Participants were then able to ‘tell us more about this’ via an open-ended text response, with no word limit imposed. Participants needed to provide an open-ended text response to this question in order to be included in this study (see Participants and Procedure section). The median length of open-text response across participants was 31 words (1–2 sentences), with a range of two (e.g. ‘not ABA’) to 661 words.

## Data management and analysis

All relevant participant data were collated into a single spreadsheet for analysis. Demographic data and participants closed-ended responses to the question about the appropriateness of early support services were analysed descriptively and presented in tabular form.

Participants’ responses to the open-ended follow-up question about the appropriateness of early support services were analysed qualitatively. Qualitative data were managed and analysed using NVivo software, following Braun and Clarke’s (2019, 2021) reflexive thematic analysis (RTA) approach. This method emphasises the active role of the researcher in interpreting the data and constructing themes, rather than treating thematic analysis as a passive, formulaic process. A constructivist epistemological framework guided the analysis as participants were understood to actively construct their own understanding of support services (Larochelle & Bednarz, 1998).

The analysis followed the six-phase process outlined by Braun and Clarke (2019), starting with familiarisation with the data, and moving through generating initial codes, creating themes, reviewing themes, defining and naming themes, and producing the final report. An inductive approach to coding data was employed, using a combination of semantic and latent approaches to analysing participant responses. This enabled interrogation of both explicit and underlying meanings, and connection to broader theory. Following coding, themes were collapsed based on their frequency and relevance to the research question, following Braun and Clarke’s (2019) guidelines.

The first author independently generated initial codes and themes, which were then refined in collaboration with CE (autistic researcher) and HW, following Braun and Clarke’s (2021) recommendation of engaging in reflexive discussions with research colleagues. While only the first author coded the full dataset, team members engaged with the data through summaries and reflective discussions to contribute to theme refinement. This process allowed for the construction of a few rich, nuanced themes that told a coherent story about participants’ perspectives.

Throughout the analysis, the first author engaged in reflexivity, documenting how personal perspectives and prior experiences might shape the interpretation of the data (Braun & Clarke, 2021). A reflective journal was maintained to trace these influences, promoting transparency in the analytic process. The analysis also considered ‘negative cases’ and diverse viewpoints. This ensured that the complexity and variability in participant experiences were thoroughly captured.

The final themes and subthemes were then supported by illustrative quotes, with participant IDs used to indicate participant group (i.e. parent, autistic adult, professional) and dataset (i.e. A or B). Thick descriptions and direct quotes were utilised to provide a rich, detailed account of the participants’ perspectives on early support services for autistic children, enhancing the trustworthiness of the findings (Tracy, 2010).

## Positionality

It is also important to note the perspectives that the research team brought to the study (Patton, 2015). Members of the research team included those with lived experience as an autistic person and/or family members of an autistic person. Some members of the team also currently, or have previously, provided support services to autistic children. All authors view autism within a neurodiversity lens, recognising that autism is a brain-based difference rather than a deficit or disorder. Some team members have previously received training or education that was based on a predominantly deficit focused, medical-model view of autism, and are actively working to change their understandings to a more affirming approach.

## Community involvement statement

Both studies from which data were drawn were co-designed, with HW, LP, and RM contributing to both projects. CE, LP, and RM are autistic adults frequently involved in conducting autism research, with LP also employed by a national autism organisation. (SP, RS, HW are family members of autistic people, and SP and HW are practitioners who currently engage in the assessment of autistic children, and/or currently or have previously provided support services to autistic children (RS). All authors were involved in the development and dissemination of the surveys, interpretation of the results and the write-up of the final study.

## Results

### Quantitative results

Participant quantitative responses regarding the appropriateness of early support services are presented in Table 1. Across the whole sample, of participants indicated ‘yes’, it was appropriate to provide early support services (50.2%), while half indicated that ‘it depends’ on the nature of those support services (48.2%). Those from Dataset A (Sulek et al., 2024) were more likely to provide an ‘it depends’ response (60.8%) than those from Dataset B (43.0%; Waddington et al., 2024). Very few participants indicated that it was not appropriate to provide early support services (*n* = 2), that they did not know whether it was appropriate (*n* = 1), or that they preferred not to say (*n* = 1).

### Qualitative results

RTA resulted in three overarching themes, with associated subthemes. These themes are ‘they are children first, after all’, ‘we shouldn’t be aiming to fix the child’, and ‘supports are beneficial’.

#### Theme 1: ‘they are children first, after all’

When considering the provision of support services to autistic children, autistic adults and parents highlighted the importance of honouring childhood, emphasising that ‘spending time with your child with autism and encouraging interaction as you would with any child, is beneficial. It does not have to be “therapy” based. They are children first, after all . . .’. (parent, A151). This included reminders that ‘if something wouldn’t be appropriate for a [neurotypical] child, it probably isn’t appropriate for an autistic child either’ (autistic individual, B79). In receiving support services, it was important to acknowledge the ‘agentic role’ children should have in their therapy, a concept that was sometimes reported to be overlooked:

We also participated in a pilot study for coaching older siblings in play skills and I noticed that the pilot therapist carefully explained to my older daughter what was going on and why and asked for her consent to keep coming – it didn’t occur to her to do the same for my [autistic] son. (parent, B24)

Like all children, autistic children should be affirmed and acknowledged for their unique identities, as one autistic parent shared, ‘it is important to send a message to young autistic people that they are okay, and they are loved and accepted for who they are’ (autistic parent, B203).

**Subtheme 1.1: ‘. . . not dominate childhood’** A common sentiment expressed by participants was the need to minimise the impact on children when providing supports, ‘. . . support services have to have minimal impact on child’s routine, and not “become an extra job” for them to meet the demands of’ (autistic parent, A86). Among this was the need to strike a balance ‘between support and being made to feel ‘different’’ (autistic individual, A47). Some participants expressed a preference for supports to be delivered in ‘the home and community where possible’ (parent, A96). Emphasising that providing support services in the home enables skill building in a ‘naturalistic, play based setting, taking into account the child’s unique strengths and interests. This does not rob children of their formative years . . .’. (autistic parent, A46). Indeed, some parents had noticed the ‘opportunity cost’ of their child engaging with clinic-based support services, meaning their child was not ‘attending regular preschool and generally he found the sessions quite tiring’ (parent, B24). As one practitioner summarised, ‘it is great to give children as many resources we can, as long as it’s not impeding on their ability to be a kid, have fun and socialise’ (practitioner, B237).

#### Theme 2: ‘we shouldn’t be aiming to fix the child . . .’

Across participants, there was a clear endorsement of neurodiversity-affirming support services aimed at helping children to ‘thrive’ (autistic individual, A30) and ‘be celebrated for their authentic self’ (autistic individual, B143), rather than those perceived to encourage children to ‘. . . “conform” or to act neurotypical’ (autistic individual, A26). As one practitioner shared, support services should be provided in ways which ‘. . .support autistic individuals [to] work towards their goals and support engagement and quality of life’, and not seek to ‘. . .change the person or reduce [their] autistic traits’ (practitioner, A110). With some participants suggesting a move away from any supports ‘. . . labeling autistic behaviors as wrong or inappropriate’ (autistic individual, A112). Participants also spoke of the need to look beyond the child, emphasising the appropriateness of ‘. . . adjusting the environment and providing accommodations to suit’ (autistic practitioner, A278) each individual. This included highlighting that ‘supporting parents, teachers and other adults around the child’ (autistic individual, B79) might lead to more enduring benefit for children compared to ‘a couple of hours’ of child directed support services. Ultimately, a combination of ‘. . . care and support provided not only to the child but also the whānau [family, Māori] as soon as possible . . .’. (practitioner, B195) might be the most effective approach to offering early support services.

**Subtheme 2.1: ‘. . .that’s going to have consequences’** It was apparent, however, that not all types of early support services were considered appropriate, with many participants sharing that those which encouraged reductions in autistic behaviours could lead to negative outcomes, ‘anything that forces masking, suppression or assimilation is likely to cause trauma’ (autistic adult, B152). One autistic practitioner shared ‘high levels of suffering’ had been caused for those clients who had engaged with non-affirming supports ‘. . . which attempted to convert them into neurotypical children . . .’. (autistic practitioner, A84) during childhood. Other participants cautioned the implementation of support services with children who were not yet able to communicate their wants and needs, ‘if they are put through certain therapies, we risk the goals and outcomes being what the parent deems important or correct and this often leads to teaching the autistic child to conform . . .’. (autistic adult, B11). Indeed, some participants expressed some guilt in selecting non-affirming support services for their child:

They also internalised that there was something ‘wrong’ with the way they engaged with the world. How I wish we had been directed to the Autistic community rather than being given the pressure-filled message that early intervention is ESSENTIAL. (parent, B318)

#### Theme 3: ‘supports are beneficial . . .’

When support services were provided in ways that prioritise childhood and celebrated the strengths inherent in autistic individuals, there was overwhelming agreement among participants that these were ‘tino pai [very good, Māori]’ (parent, A135). One autistic adult, reflecting on their experience of childhood support services, shared that ‘having access to therapies allowed me to explore my world and grow. . ’. (autistic individual, A103). Providing support services during childhood was believed to optimise ‘. . .quality of life outcomes for children and their families and in building capacity of support systems (early childhood educators and school staff)’ (practitioner, A160). For one parent, accessing early supports meant ‘. . .teaching [the child] how to help herself and teach her that it’s ok she needs extra or different ways of doing things helps everyone in her circles’ (parent, B14). For another parent, the support their child received paved the way for improved communication, as they expressed, ‘therapy is so gently done with our daughter, and it has helped her so much to non-verbally communicate and navigate a little easier through life’ (parent, B62). Recognising that support services had helped them become ‘who I am today’, one autistic participant highlighted that services which meet child needs ‘. . .can be really beneficial to help them blossom into who they are’ (autistic individual, B143).

**Subtheme 3.1: ‘. . .appropriate for the child, their family, and their situation’** Although participants generally agreed that there were benefits to be gained from autistic children accessing support services, it was emphasised that these should be ‘highly individualised’ (parent, A313). This was thought to be particularly important given the diversity inherent in autism, ‘autism is a spectrum so it would depend highly on where the child sits on that spectrum from needing social/relationship building skills, to speech development and therapy, to other various needs. It would really depend on the child’ (parent, B42). Alignment with child and family goals was also thought to be essential, as expressed by one practitioner, ‘I am interested in providing supports that reflect family priorities or goals and provide functional benefit/ support a child to engage in their natural environment’ (practitioner, B115). For some families, it was a balancing act to find the best fit between the most appropriate support for the child and the family context, with some parents feeling pressured ‘. . .to become experts in psychology, body language, social interaction’, which added a burden on ‘already stressed parents’ (autistic parent, B123). With another autistic parent highlighting that the ‘homework’ provided by her child’s therapist was often left incomplete ‘. . . as it seemed a lot and didn’t fit into executing else that was going on . . .’. (autistic parent, B140). As one practitioner who was also the parent of an autistic child shared, ‘. . .it is very appropriate to provide supports for those who would like to receive them’ (practitioner and parent, B38).

**Subtheme 3.2: ‘the earlier. . . the better’** When contemplating the suitability of providing support services to autistic children, the timing of support provision was regarded as a crucial factor to consider. Participants largely agreed that providing support services during early childhood coincide with an ‘. . . important phase of their development and they would be left behind without this’ (Parent, A141). With another parent noting ‘so much critical social, emotional and physical development happens under the age of 5, therefore professional supports at this age are critical’ (parent, B176). As one practitioner shared, benefits of early support services also extend beyond those experienced by the child themselves, ‘the earl[ier] support is put in place the more it benefits a child. It is also extremely important to both teach and support a child’s family around autism’ (practitioner, B75).

Some autistic participants further underscored the importance of early support services, indicating that there might be missed opportunities or disadvantage associated with not receiving support when needs were initially identified:

I had developmental issues that showed up in my 3 year old early childhood exam, but these were ignored. I couldn’t tie my shoe laces until year 6 and as behind my peers in daily living skills (and still am). If my treatment had commenced at age 3 or 4 my life may be different. (autistic individual, A101)

With another autistic participant highlighting that this might be most apparent for young girls who do not receive a diagnosis until later in life:

Personally if I was diagnosed being on the spectrum earlier it would’ve lessened the torment during school and high school so having support would be most effective as girls wouldn’t feel so bad about themselves and would have help when feeling being on the wrong path in life. (autistic individual, A153)

However, it is important to note that not all participants agreed that earlier means better, as participant shared, ‘I believe that had he received support too early during his primary school years, he would not have enjoyed his childhood and unstructured play, which I consider was very important for learning too’ (parent and practitioner, A196). In the end, participants identified that prioritising early identification and support services that allow autistic children ‘. . . to live with dignity and as independently as possible’ (autistic individual, B71) should be the primary focus.

## Discussion

This study aimed to provide a current understanding of stakeholder perspectives regarding the appropriateness of early support services provided to autistic children. Quantitative results indicate that participants were relatively evenly split between believing that early support services were appropriate, and that the appropriateness depended on the nature of the support service. We used RTA of qualitative responses to explain the reasons underlying these varying perceptions. This resulted in three themes which included an emphasis on honouring the child and childhood, the importance of strengths-based and neurodiversity-affirming support services, and the benefits that appropriate support services can provide.

The significance of minimising disruptions to the experience of childhood is broadly consistent with Trembath et al.’s (2021) evidence-based framework for determining the optimal amount of support for autistic children. The authors emphasise that, when deciding on the amount of support, practitioners should account for the extent to which support services are a priority for the family and can be feasibly delivered (i.e. their practicality). They further propose that practitioners should balance the possible benefits and costs of the proposed amount of support, including the risk of reducing the time the child spends in preferred activities (i.e. their defensibility). Providing support at the cost of autistic individuals being able to engage with other important life activities has also been highlighted by autistic advocates (Autistic Self Advocacy Network, 2021) as a ‘red flag’.

The importance of allowing children to be active agents in the decisions that concern them is echoed by autistic advocates and is enshrined in international laws. Specifically, advocates emphasise the rights of autistic individuals to be informed about the support services they access and to be actively involved in setting their own goals, as appropriate (Autistic Self Advocacy Network, 2021; Leaf et al., 2022; Schuck et al., 2022). Article 12 of the United Nations Convention on the Rights of the Child (1989) also states that every child has the right to freely express their views on all matters affecting them. There are, however, practical issues in realising this right. More research is needed to determine the most effective ways to ensure that autistic children’s desires surrounding support services are not only heard but also enacted by the adults who support them. This is particularly true for young children and those who communicate using ways other than speech.

While this research highlighted the appropriateness of neurodiversity-affirming early support services across all participant groups, this was overwhelmingly apparent among autistic participants, including those who also identified as both parents and professionals. In the context of the broader neurodiversity movement, this is perhaps unsurprising, as autistic advocates have been key drivers for change in this space since the movement began in the 1990s (Chamak et al., 2008; Gillespie-Lynch et al., 2017). However, in line with emerging research (see Dawson et al., 2022; Schuck et al., 2022; Waddington et al., 2024), we did find evidence of an increasing commitment to neurodiversity-affirming approaches echoed by non-autistic individuals in this sample. In describing neurodiversity-affirming support services, participants highlighted these should promote greater inclusion and autistic quality of life, rather than trying to reduce characteristics of autism. With some participants further emphasising the need to look beyond approaches which directly target autistic individuals themselves, and instead focus on creating more supportive and accessible environments. This is largely consistent with the desired characteristics of support services, as described by neurodiversity researchers and advocates (Autistic Self Advocacy Network, 2021; Dawson et al., 2022; Gillespie-Lynch et al., 2017; Pellicano & den Houting, 2022). This does raise questions about the appropriateness of many available early support services, particularly those which focus on a reduction in autism characteristics.

The third theme outlined the possible benefits that could be conferred by accessing early support services. Some participants stressed, however, that these services must align with child and family goals and acknowledge individual differences. This general endorsement of early support services, with caveats from some participants, helps to reconcile the split between participants who quantitatively indicated that early support was appropriate and those who expressed that it depended on the nature of the supports. Of note was the general endorsement by all participant groups of appropriate support services provided early, with agreement that this would result in better outcomes for children. This is unsurprising given the pervasive messaging often communicated to families following their child’s diagnosis of autism regarding the need for early, and often intensive, support services to optimise child outcomes (Edwards et al., 2017; Leaf et al., 2022). This messaging persists despite the variable level of empirical support for available approaches, including for whom they are most effective (Bottema-Beutel, 2023; Trembath et al., 2023). Participants also highlighted the potential risks of not providing appropriate support services, in particular for young girls. Research has shown that females are less likely to receive a diagnosis during childhood, despite apparent similarities to males in their expression of autism characteristics in the early years (Chellew et al., 2024). Given suggested links between masking and camouflaging, and poorer mental health outcomes (Cook et al., 2021), missed opportunities to provide support services during the early years might have a particularly negative impact on females, who are often diagnosed later in life.

The findings across themes broadly align with frameworks for understanding what a ‘good life’ should look like for autistic people, a key aim of neurodiversity-affirming support services. For example, Nussbaum’s (2000) capabilities approach describes the 10 core elements of a ‘thriving human life’ and has previously been applied to research on autistic adulthood (Pellicano & Heyworth, 2023). This approach also relates to children and families within this study. For example, core elements such as ‘senses, imagination, and thought’ and ‘play’ are highly relevant to the first theme: ‘they are children first, after all’. Particularly, participants emphasised the need for the child to have access to enjoyable experiences and activities and to be free from pain and distress. Another core element is ‘control over one’s environment’ which relates to the need to actively involve children in decisions around support services. Finally, the core element of ‘affiliation’ includes the need to be treated as a ‘dignified being’. Participants in this study spoke of the need to create more accessible environments and to help those around that child, such as parents and teachers, to better understand them. Quality-of-life frameworks, such as Nussbaum’s (2000) capabilities approach may be useful determining how to genuinely improve autistic people’s lives.

Strengths of the study include the reasonable sample size spanning a range of key stakeholder groups. We also acknowledge limitations in the study design, particularly the drawbacks inherent in utilising open-ended survey data in this qualitative analysis. Although participants were not given a character limit when providing their responses, the survey format limited our capacity to clarify any comments made or further unpack participant responses. While we were still able to collect a comprehensive range and depth of opinions regarding the appropriateness of early support services, future research would benefit from utilising methodologies which enable a deeper exploration of participant perspectives. As noted earlier, there was a minor difference in the wording of the early support services quantitative survey question across surveys, specifically that one referred to support services for *preschool aged children* and the other for *autistic children (aged between 0 and 12* *years)*. This might have led to differences in participant responses, however, support for the reported themes and subthemes were drawn from both datasets. It is also important to note that responses to the qualitative question analysed here were not required of participants when completing the survey, however, 54% and 40% of the broader project samples chose to elaborate on their views regarding early support services, including representation across all key stakeholder groups. Furthermore, it is possible that those who provided responses to the qualitative question were generally more supportive of early support services. Participants in this study were recruited through autism-related social media groups and the authors’ own networks. Many of these groups explicitly promote neurodiversity-affirming approaches and the authors view autism through a neurodiversity-affirming lens. As such, the participants in this research may have been more likely to endorse neurodiversity-affirming views. We had a reasonable representation from autistic individuals in this sample (40.3%); however, we did not recruit autistic children and would encourage future research to employ developmentally appropriate methodologies to seek the perspectives of autistic children themselves.

## Conclusion

This research underscores critical need to exercise caution in the selection and implementation of early supports services, particularly where these might seek to normalise autistic children. The results emphasise the importance of support services which cultivate meaningful life skills, quality of life, self-determination, and self-esteem, and advocate for a paradigm shift towards tailored support services which prioritise the unique strengths and needs of each autistic child. Collaborating with parents, who serve as decision-makers for their autistic children, and involving the children themselves in the decision-making process is paramount. Balancing these considerations can be complex, but there appears to be generalised agreement among key stakeholders that appropriate, respectful support can lead to positive outcomes for both children and their families.
